# Intelligence

**Published:** 1833-04

**Authors:** 


### INTELLIGENCE.

### ST. GEORGE'S HOSPITAL.

Dr. Macleod has been appointed one of the physicians to this hospital, *vice* Dr. Hewitt, resigned. When the vacancy was first declared, some other candidates started; but it was quickly discovered that opposition would be in vain, and, consequently, Dr. Macleod obtained the important office without the usual trouble of a contested election.

### MIDDLESEX HOSPITAL.

After an arduous contest, Mr. Tuson has been elected assistant surgeon to this institution, in the room of Mr. Arnott, who succeeded to the surgeoncy, without opposition, upon the demise of Mr. Joberns. Mr. Tuson was opposed by Mr. Shaw and Mr. Phillips, and his majority over the former of these gentlemen was only eighteen.

NEW MEDICAL SOCIETY AT THE MIDDLESEX HOSPITAL.

A new society has recently been formed by the pupils of this hospital, which is to meet every Tuesday evening, at eight o'clock. The cases in the hospital will at all times furnish the members with ample food for discussion, and, in our opinion, they will do well to confine themselves to the consideration of the cases they have immediately under their eyes, rather than to follow the example of some other societies of a similar nature, in which much time is occupied, and perhaps we might say wasted, by visionary speculations, and proposals for new modes of treatment. We believe that the physicians and surgeons of the hospital will alternately preside.

HONOURABLE DISTINCTION CONFERRED UPON SIR ASTLEY COOPER.

The King of France has bestowed upon Sir Astley Cooper the decoration of the royal legion of honour. It is said that this mark of distinction was conferred upon Sir Astley at the suggestion of Prince Talleyrand. From the general and well-merited esteem in which Sir A. is held in this country, we are sure that all his professional brethren will feel gratified at the mark of attention which has thus been bestowed by a foreign monarch.

Dr. Robert Brown, the celebrated naturalist and botanist, has been elected foreign member, in the place of the lamented Scarpa, by the Academy of Sciences in Paris. Many men of science and strong claims to this honour were nominated as candidates, but Dr. Brown had a considerable majority of votes.

THE COMPANY OF APOTHECARIES *v.* COLLINS, AN EDINBURGH PHYSICIAN.

In this case the defendant was prosecuted by the Apothecaries' Company for practising pharmacy in England, without a licence. For the defence it was urged, that the defendant had received the degree of doctor of medicine in Scotland. It was ruled by the judge, that he was nevertheless amenable to the "Apothecaries' Act," and he was sentenced to pay a fine of £20.—A petition from the medical students of the university of Edinburgh has, we understand, been transmitted to parliament, praying for the abolition of the Apothecaries' Act, which precludes them, even when admitted as licentiates of the College of Physicians or Surgeons of Edinburgh, from dispensing medicines in England.

A ZEALOT IN THE HOMŒOPATHIC DOCTRINES.

We have received a most extraordinary epistle from one John Borthwick Gilchrist; but whether our correspondent has dipped his pen in a sportive mood, or whether he is in downright earnest, we cannot venture to determine. If he be inclined to be merry, we have no objection to laugh *with* him for a moment, by way of distraction from our ordinary and graver duties; and if he be really serious, why then, in spite of our editorial gravity, we must laugh *at* him.

We must premise our brief account of Mr. G.'s letter, by declaring that we mean not to venture any dogmatic opinion upon the merits of the homœopathic method of treatment: we know too little of it to be competent judges of its value, but we may just hint our belief that it is a kind of do-nothing and procrastinating system, under which, no doubt, patients frequently recover, because nature was all-sufficient for their cure. But to the epistle before us, of which, however, we can only extract the most pithy parts.

It is addressed to "all the practitioners of the medical, medicinal, and mechanical branches of the healing art, in every quarter of the world." Mr. G. tells us "that he was himself educated for all the above-mentioned departments of our useful vocation," and, consequently, that none of his brethren can imagine his address "is intended to convey the smallest offence, either in our collective or individual capacities." Any such proemial assurance was quite unnecessary, for his letter is not at all calculated to excite the slightest feeling of displeasure towards the writer. He must, we think, be regarded as "a merry soul," but a little too tedious in his fun. "While still a young man," Mr. G. began to have certain misgivings as to the solidity and efficacy of the medical doctrines of the day, and the "very suspicion of something unsound in the foundation of medical science," together with a large share of tender-heartedness, which totally unfitted him for the melancholy scenes incidental to the profession of physic, induced him to relinquish a business, from which he "could hardly expect either fame or fortune," for "others of a literary nature, more congenial with his own feelings," and under the East India Company's protection. "Having thus devoted the prime of youth and manhood of his existence" to other avocations, all his medical cunning was lost, except "a few elemental reminiscences," which we are informed have been very useful to him during a long season of personal suffering. He expresses the philanthropic wish that he may still live "until the public shall reap the whole of the advantages proposed to be derived by mankind at large through the medium of this earnest appeal," the object of which is to point out the very wonderful effects of "a new system of physic," which is no other than that of which we have all heard, namely, Dr. Hahnemann's, or the Homœopathic Doctrine.

Mr. Gilchrist is mistaken in supposing it necessary to give a particular description of the creed which he so warmly espouses. Every body knows, as well as he does, the peculiarities of the Hahnemannian belief, although few, perhaps, believe so implicitly in their rationality. There may, however, for aught we can declare to the contrary, be some very worldly motive in informing us precisely at whose shops the "homœopathic publications" may be purchased; but, as we are very unwilling to stop even the transfer of a sixpence to those honest worthies the booksellers, we forbear from descanting upon the probable motives that have induced Mr. G. so particularly to specify their "whereabouts."

Come we now to some of the sober eulogies in which John Borthwick Gilchrist indulges. We shall mark his phrases by inverted commas, and only take the liberty of interposing here and there a connecting link, when we wish, like the schoolboy, to skip a little, and cut short a rather long and weary task. "Gratitude," says J. B. G., "bids me hail the new system as the gospel of medical salvation, whose genial balm of Gilead may be collected from Hahnemann's organon, which I at least may justly call the New Testament of my medical faith." It appears; that for "six revolving summers" Mr. G. was "under the hands of very celebrated and generous Allopathists, but all in vain:" in as many weeks, the "Homœopathic remedies acted like a spell, though the dose taken, once in eight days, is not so much as a very moderate pinch of snuff," and, "from a constant state of low spirits, he became all at once as light and gay in body and mind as a skylark," and now "feels himself hourly soaring on the pleasures of hope!" being rescued from "a long string of horrors, nicknamed *blue devils*." Mr. G. assures us, that "how, when, where, and why" the potent little doses act, he cannot tell. He never saw the physician who prescribed for him. Every thing was affected by letter; the medicine was sent by post; and all he knows of it is, that "its nature is antisporic, the colour white, the taste sweetish, its bulk a pinch of snuff," and that it is to be taken once a week. Such were the happy effects of the "sweet, white pinch," that, after "being virtually for several years buried alive, for the most essential purposes of existence," Mr. G. "actually feels like a man risen from the dead," and, "instead of being still charged with gloomy misanthropy, his whole frame glows with the most expansive benevolence," and he "could, with delight, *if able to do so*, fly on the wings of the morning, or, like the fearless eagle, from east to west, and north to south, that he might enjoy the supreme felicity of making all the sick well, and all the sad happy." "As one proof of boundless philanthropy, I hereby (concludes our correspondent) cheerfully forget and forgive every offence which any person has ever committed against me; and I moreover conjure whomsoever I may have offended, to evince the same charitable disposition towards me."

So much for the effects of the homœopathic treatment upon Mr. J. B. Gilchrist. From the letter itself, we certainly should never have imagined that he had jumped from physic to literary pursuits, if he had not very kindly so informed us; and, as his punctuation and style of writing are any thing but sound, we advise him to take another "pinch" of the sweet white powder; for who knows but that the remedy may cure the maladies of his pen, as it has those of his body.

2

### LITERARY INTELLIGENCE.

We have been favored with a sight of a very ingenious and useful work, which will soon be published by Mr. SPRATT, entitled "Obstetric Tables," the object of which is to illustrate elementary works on midwifery. It will contain twelve coloured plates, representing in layers the anatomy of the parts engaged in labour, and also the various positions of the fœtus, and the mode in which instruments are to be applied. The price is moderate; and we think it cannot fail to be successful.

Preparing for publication, A Translation of the Practical Treatise on the Diseases of the Uterus and the Appendages, of Mme. BOIVIN and M. DUGES, by G. O. HEMING, of Kentish Town, Member of the Royal College of Surgeons; with additions.

### METEOROLOGICAL REGISTER,

By Messrs. Harris and Co., Mathematical Instrument Makers, 50, High Holborn.

| | Moon. | Rain gauge. | Thermometer. | | | Barometer. | | De Luc's Hygrometer | | Winds. | | Atmospheric Variation. | | |
|---|---|---|---|---|---|---|---|---|---|---|---|---|---|---|
| Feb. | | | 9 a.m. | max. | min. | 9a.m. | 10p.m. | 9a.m. | 10p.m. | 9 a.m. | 10p.m. | 9 a.m. | 2 p.m. | 10 p.m. |
| 20 | | | 43 | 45 | 40 | 28.86 | 29.04 | 76 | 77 | WSW | NW | showery | cloudy | rain |
| 21 | | .11 | 42 | 46 | 39 | 29.57 | .80 | 80 | 82 | NE | E | overcast | showery | cloudy |
| 22 | | | 43 | 45 | 36 | .80 | .80 | 82 | 81 | ENE | E | fine | fine | fine |
| 23 | | | 38 | 43 | 35 | .67 | .58 | 79 | 76 | ENE | ESE | | | |
| 24 | | | 40 | 46 | 38 | .51 | .46 | 79 | 83 | SE | S | rain | rain | cloudy |
| 25 | | .06 | 42 | 47 | 43 | .40 | .35 | 82 | 82 | WSW | SW | foggy | cloudy | fine |
| 26 | | | 46 | 50 | 41 | .15 | .96 | 83 | 82 | SSW | SSE | showery | | |
| 27 | ☽ | | 43 | 46 | 39 | 28.91 | 29.06 | 82 | 81 | S | NW | rain | cloudy | |
| 28 | | .22 | 42 | 45 | 38 | 29.09 | 28.83 | 83 | 79 | SW | W | foggy | rain | |
| Mar. | | | | | | | | | | | | | | |
| 1 | | | 43 | 47 | 38 | 28.87 | 29.13 | 77 | 75 | WSW | WNW | fine | fine | |
| 2 | | | 45 | 50 | 48 | 29.53 | .66 | 75 | 76 | WNW | W | | | |
| 3 | | | 51 | 54 | 47 | .68 | .46 | 78 | 76 | SW | SW | cloudy | fine | cloudy |
| 4 | | | 50 | 55 | 42 | .51 | .67 | 75 | 75 | SSE | SE | foggy | fine | cloudy |
| 5 | | | 44 | 51 | 42 | .80 | .80 | 75 | 74 | S | N | | | |
| 6 | ○ | | 44 | 46 | 38 | .92 | .97 | 74 | 71 | NNE | N | fine | | |
| 7 | | | 41 | 45 | 34 | 30.04 | 30.12 | 74 | 71 | N | E | | cloudy | |
| 8 | | | 38 | 40 | 31 | .07 | .07 | 69 | 70 | E | E | cloudy | fine | cloudy |
| 9 | | | 37 | 39 | 34 | 29.92 | 29.82 | 72 | 72 | NNE | ENE | snow | | cloudy |
| 10 | | | 38 | 41 | 32 | .82 | .82 | 73 | 73 | NE | NE | fine | | fine |
| 11 | | | 36 | 40 | 34 | .84 | .87 | 73 | 72 | E | E | snow | cloudy | cloudy |
| 12 | | | 37 | 41 | 32 | .83 | .77 | 69 | 68 | E | E | fine | fine | fine |
| 13 | ☾ | | 34 | 40 | 29 | .57 | .35 | 67 | 68 | ESE | SSE | haily | | |
| 14 | | | 35 | 42 | 32 | .21 | .21 | 68 | 66 | SSE | SE | fine | | |
| 15 | | | 34 | 40 | 29 | .21 | .29 | 66 | 71 | ESE | E | | | |
| 16 | | | 43 | 48 | 36 | .29 | .32 | 74 | 73 | ESE | ESE | | | |
| 17 | | | 39 | 43 | 38 | .35 | .44 | 80 | 86 | E | E | showery | rain | rain |
| 18 | | .48 | 40 | 42 | 35 | .53 | .61 | 88 | 85 | E | E | | fine | fine |
| 19 | | | 39 | 45 | 32 | .77 | .81 | 83 | 79 | ENE | ENE | fine | showery | fine |

The quantity of Rain fallen in February, was 1 inch and 7.100ths.

